# Satisfaction with surgical correction of stress urinary incontinence in women: A pilot study in Almaty, Kazakhstan

**DOI:** 10.5195/cajgh.2014.142

**Published:** 2014-08-08

**Authors:** Sayara M. Mukhtarova, Gulzhahan K. Omarova, Aynura I. Yoldasheva

**Affiliations:** Kazakh National Medical University, Almaty, Kazakhstan

## Introduction

Stress urinary incontinence (SUI) is a very important problem in the fields of gynecology and geriatrics. While SUI in not necessarily related to elevated mortality, it leads to significant loss of quality of life, psychological distress, and social isolation or avoidance of recreational activities. 1 Little has been published on SUI in Kazakhstan and the Central Asian region.

According to the International Continence Society, urinary incontinence is the “involuntary loss of urine that is objectively demonstrable and a social or hygienic problem”. SUI is the most common form of incontinence. SUI occurs when there is involuntary leakage of urine in the event of sudden extra pressure (‘stress’) on the bladder, often associated with physical activity. The key reason for incontinence development is weakened pelvic floor muscles, which support the bladder and urethra. 2

According to international statistics, about 24% of women aged 30 to 60 years, and more than 40% of postmenopausal women reported symptoms of SUI. 2 According to the United States and European publications, 45% of women aged 40 – 60 years suffer stress incontinence, and in Russia, 38%. 3,4 SUI affects women of all ages - young mothers, women in pre- and perimenopausal age groups, and the elderly. It is important to note that SUI is not an inevitable part of aging. There are multiple surgery types to correct SUI that are currently being practiced in Kazakhstan, including injectable bulking agents, retropubic colposuspension, sling procedure, and many others. Little has been published about patient satisfaction with those procedures in Central Asian region.

Thus, the aim of this pilot investigation was to examine the risk factors for stress incontinence in women who were seeking surgical correction for stress incontinence. The secondary goal of our investigation was to evaluation satisfaction with surgical procedures to correct SUI 3, 6, and 12 months after surgery.

## Methods

This study included 40 women with stress incontinence treated in Almaty, Kazakhstan. These women underwent traditional surgery (Burch Colposuspension) and combined methods using synthetic endoprostheses (Tension-free slings (TVT) with the plastic of the perineum, vaginal hysterectomy + TVT). Satisfaction with the surgical procedure was assessed 3, 6, and 12 months after surgery. Data were obtained through medical record review, as well as by collecting self reported satisfaction after surgical treatment. Patients were recruited from two medical facilities: City Hospitals #1 and #7, Almaty, Kazakhstan.

## Results

The average age of the patients in this pilot study was 52.9 ± 1.6 years, with 20% peak and late reproductive stage, 30% perimenopausal, and 50% postmenopausal. Reproductive age cut offs are based on the staging system outlined by Harlow *et al*. 5 Evaluation of reproductive history of patients revealed that all women had a history of childbirth: 10 (25%) - one birth, 24(60%) - up to three births, and in 6 (15%) - four or more. In this sample, 36 women (90%) delivered newborns of normal body weight, whereas four women (10%) had newborns with macrosomia. Obstetric injuries were reported in 42.5% of cases. Gynecological morbidity analysis showed that 35 (87.5%) had comorbid gynecologic pathologies including uterine fibroids (18 (45%)), cervical erosion (10 (25%), adenomyosis (3 (7.5%)), disease of the ovaries and fallopian tubes (3 (7.5%)), and Bartholin’s gland cyst (1 (2.5%)).

Descriptive statistics demonstrated that the major risk factors for stress urinary incontinence in this sample of women, were a combination of 2 or more factors, including: hypoestrogenia in 20 women (50%), obstetrical trauma in 17 women (42.5%), increased abdominal pressure in 6 (15%), complicated labor in 6 (15%), and connective tissue dysplasia in 4 (10%).

We found that 28 (70%) of patients in this group were overweight or obese. Specifically, 10 (25%) were classified as Class 1 obese (BMI 30 – 34.9 kg/m^2^), 18 (45%) were overweight (BMI 25 – 29.9 kg/m^2^), and 12 (30%) were of normal weight (BMI 18.5 – 24.9 kg/m^2^). Gynecologic health assessment showed that 35 (87.5%) patients had comorbid gynecologic conditions including uterine fibroids, cervical erosion, and ademomyosis.

Overall, we found that women were satisfied with symptoms post surgery. Significant improvement and/or absence of complaints after 3 months was found in 34 (85%) women, after 6 months - in 35 (87.5%), and after 1 year - in 37 (92.5%).

## Conclusion

Our pilot study demonstrated that SUI occurs most frequently in parous women, mainly in the postmenopausal period. Our results are consistent with previously published evidence about the risk factors of SUI. It should be noted that previous studies reported more frequent incidence of macrosomia than in our study. 2,3 Previous publications also suggest that risk factors for the development of SUI include conditions such as hypoestrogenia, heavy physical work, varicose disease, and hernias of different locations, which were all more common in previous publicactions than in the current study. 2,3

SUI in women is a polyethiologic disease and is directly related to age, parity, and metabolic disorders. Surgical correction appears to be effective procedure in our sample, with 92% women reporting satisfaction with procedure 1 year after the surgery. Various treatment options for this condition, including innovative options such as global postural re-education, must be considered globally, especially in settings with limited resources. 6
